# GPDBN: deep bilinear network integrating both genomic data and pathological images for breast cancer prognosis prediction

**DOI:** 10.1093/bioinformatics/btab185

**Published:** 2021-03-18

**Authors:** Zhiqin Wang, Ruiqing Li, Minghui Wang, Ao Li

**Affiliations:** School of Information Science and Technology, University of Science and Technology of China, Hefei AH230027, China; School of Information Science and Technology, University of Science and Technology of China, Hefei AH230027, China; School of Information Science and Technology, University of Science and Technology of China, Hefei AH230027, China; Centers for Biomedical Engineering, University of Science and Technology of China, Hefei AH230027, China; School of Information Science and Technology, University of Science and Technology of China, Hefei AH230027, China; Centers for Biomedical Engineering, University of Science and Technology of China, Hefei AH230027, China

## Abstract

**Motivation:**

Breast cancer is a very heterogeneous disease and there is an urgent need to design computational methods that can accurately predict the prognosis of breast cancer for appropriate therapeutic regime. Recently, deep learning-based methods have achieved great success in prognosis prediction, but many of them directly combine features from different modalities that may ignore the complex inter-modality relations. In addition, existing deep learning-based methods do not take intra-modality relations into consideration that are also beneficial to prognosis prediction. Therefore, it is of great importance to develop a deep learning-based method that can take advantage of the complementary information between intra-modality and inter-modality by integrating data from different modalities for more accurate prognosis prediction of breast cancer.

**Results:**

We present a novel unified framework named genomic and pathological deep bilinear network (GPDBN) for prognosis prediction of breast cancer by effectively integrating both genomic data and pathological images. In GPDBN, an inter-modality bilinear feature encoding module is proposed to model complex inter-modality relations for fully exploiting intrinsic relationship of the features across different modalities. Meanwhile, intra-modality relations that are also beneficial to prognosis prediction, are captured by two intra-modality bilinear feature encoding modules. Moreover, to take advantage of the complementary information between inter-modality and intra-modality relations, GPDBN further combines the inter- and intra-modality bilinear features by using a multi-layer deep neural network for final prognosis prediction. Comprehensive experiment results demonstrate that the proposed GPDBN significantly improves the performance of breast cancer prognosis prediction and compares favorably with existing methods.

**Availabilityand implementation:**

GPDBN is freely available at https://github.com/isfj/GPDBN.

**Supplementary information:**

Supplementary data are available at *Bioinformatics* online.

## 1 Introduction

The most common malignancy among females is breast cancer, which is one of the leading causes of cancer-related deaths in the world (Hortobagyi *et al.*, 2005). As reported by WHO, more than 1.3 million new cases of breast cancer are diagnosed, and the death toll is as high as 458 000 each year (Nguyen *et al.*, 2013). As a very heterogeneous disease, breast cancer has been reported with distinct prognoses on the basis of morphological and molecular stratifications (Huang *et al.*, 2019), which represents the major hurdle for accurate diagnosis and curative therapy. Therefore, there is an urgent need to design computational methods to accurately predict the prognosis of breast cancer, which may further assist clinicians to prescribe the most appropriate therapeutic regime (Sun *et al.*, 2018a,b).

There have been many studies in predicting the prognosis of breast cancer that assign breast cancer patients into a poor and a good prognosis group based on genomic data and/or pathological images (Gevaert *et al.*, 2006; Sun *et al.*, 2018a,b; Yu *et al.*, 2016). Genomic data, especially gene expression profiles (Xu *et al.*, 2012) obtained from high-throughput platforms, has been widely adopted in breast cancer prognosis prediction (Moon *et al.*, 2017; Sun *et al.*, 2018a,b; Wang *et al.*, 2005). The initial studies aim to discover genes that can separate patients with good prognosis from those with poor prognosis. After that, there are plenty of studies generate a multigene predictor based on a hypothesis developed from in vivo or in vitro experiments and then apply it to breast cancer samples (Reis-Filho and Pusztai, 2011). For example, Van De Vijver *et al.* (2002) recognize a 70-gene prognostic signature from 117 patients with breast cancer by correlating candidate genes with the outcome and determine the optimal set of genes by using leave-one-out cross validation. Wang et al. (2005) further identify a 76-gene signature representing a strong prognostic factor that can be applied to identify patients who have a favorable prognosis. These studies undoubtedly have not only contributed to our understanding of the heterogeneity and complexity of breast cancer behavior but also provided computational methods to distinguish between patients with good and poor prognosis.

Besides aforementioned prognosis prediction methods using genomic data, the continued importance of pathological analysis of tumors should be emphasized, which has been confirmed to provide independent prognostic information of breast cancer (Reis-Filho and Pusztai, 2011). With the emergence of digital whole-slide images, comprehensive computational methods for pathological image analysis have demonstrated promising capability to improve efficiency, accuracy and consistency compared with human examination (Cheng *et al.*, 2017; Courtiol *et al.*, 2019). With rapid growth of computing resources, many computational pathological methods have been proposed for predicting the prognosis of breast cancer (Xu *et al.*, 2016) and a considerable number of other cancers such as lung (Yu *et al.*, 2016; Zhu *et al.*, 2016) and kidney (Cheng *et al.*, 2018) cancers. In this way, hundreds of pathological image features, characterizing cell size, shape, distribution and texture of nuclei, can be extracted from pathological images, which can provide important prognostic information for further investigation.

In addition to aforementioned studies using either genomic data or pathological images, there is an increasing interest in combining both of them that may contribute to more accurate cancer prognosis prediction (Cheng *et al.*, 2017; Ning *et al.*, 2020; Shao *et al.*, 2020). With the fast development of computer-aided technology, machine learning methods are widely applied to predict cancer prognosis. For example, by using SVM with a Gaussian radial basis kernel, Yuan et al. (2012) integrate both genomic data and pathological images to improve the performance of breast cancer prognosis prediction. Sun et al. (2018a,b) develop a multiple kernel learning approach named GPMKL, which employs heterogeneous features extracted from genomic data and pathological images. Besides breast cancer, Cheng et al. (2017) predict the survival outcomes of renal cell carcinoma patients by combining quantitative image features derived from pathological images and eigengenes derived from gene expression profiles. These studies clearly show that genomic data and pathological images are complementary to each other and can acquire better performance of prognosis of patients when employed together.

Recently, deep learning-based approaches for the integration of data from different modalities have been proposed (Ngiam *et al.*, 2011) and successfully applied in cancer prognosis prediction (Cheerla and Gevaert, 2019; Mobadersany *et al.*, 2018; Yao *et al.*, 2017), which are highly flexible and can interpret the complexity of data in a non-linear manner (Huang *et al.*, 2019). For example, Mobadersany et al. (2018) develop a unified framework named genomic survival convolutional neural network (GSCNN) to predict time-to-event outcomes of patients diagnosed with glioma. To derive highly predictive prognostic features, GSCNN incorporate features embedded within pathological images with genomic features by feeding them into a multi-layer neural network that can add additional non-linear transformations to extracted features. Although the prediction accuracy of GSCNN exceeds human experts according to the current clinical standard, the direct combination of image and genomic features in GSCNN is suboptimal as it may ignore the intrinsic relationship of the features across different modalities (Shao *et al.*, 2019; Yao *et al.*, 2017). Alternatively, Yao et al. (2017) introduce an efficient correlation loss function for training deep learning-based survival model by explicitly maximizing the correlation among image and genomic features, and report quite promising performances for lung and brain cancer. Also, Cheerla and Gevaert (2019) exploit similarities between image and genomic features from the same patient via maximizing a cosine similarity-based loss function. The success of these studies highlights the importance of developing sophisticated deep learning-based methods for more accurate breast cancer prognosis prediction by efficiently leveraging both genomic data and pathological images.

In this study, we propose a novel unified framework named genomic and pathological deep bilinear network (GPDBN) for integrating genomic data and pathological images to predict breast cancer prognosis. Our GPDBN framework includes an inter-modality bilinear feature encoding module (Inter-BFEM) and two intra-modality bilinear feature encoding modules (Intra-BFEMs) to achieve effective information between and within the genomic data and pathological images. Given pre-processed genomic data and pathological images, Inter-BFEM generates inter-modality bilinear features to model complex inter-modality relations for fully exploiting intrinsic relationship of the features across different modalities. In addition, Intra-BFEMs calculate the intra-modality bilinear features within each modality for capturing intra-modality relations. We argue that the intra-modality relations play an important role for breast cancer prognosis prediction, as they can provide valuable information complementary to that included in inter-modality relations (Gao *et al.*, 2019). Therefore, GPDBN further combines the intra- and inter-modality bilinear features by using a multi-layer deep neural network. The experimental results verify that our proposed GPDBN framework enhances the performance of breast cancer prognosis prediction and outperforms existing approaches using both genomic data and pathological images.

## 2 Materials and methods

### 2.1 Dataset and pre-process

Breast cancer patient samples adopted in this study include matched digital whole-slide images and gene expression profiles (totally 20 436 genes), which are acquired from the Cancer Genome Atlas (TCGA) data portal (Zhu *et al.*, 2014). By following previous work (Cheng *et al.*, 2017), patients with missing follow-up are excluded and finally 345 patients are enrolled in this study. These patients are further categorized into longer-term or shorter-term survivors by the criterion of 5-year survival (Gevaert *et al.*, 2006; Nguyen *et al.*, 2013). Accordingly, shorter-term survivors are labeled as 1 (i.e. poor prognosis) while the longer-term survivors are labeled as 0 (i.e. good prognosis). In consistent with previous prognosis prediction studies (Ching *et al.*, 2018; Huang *et al.*, 2019), 5-fold cross-validation is conducted on the dataset with 80% of the data in each fold used for training and 20% for testing.

Similar to Ding et al. (2016), the genes with missing values (NA) in more than 10% patients are deleted. After that, gene expression profiles with 19 006 genes are normalized with *z*-score by standardizing the distribution and then discretized into under-expression (-1), over-expression (1) and baseline (0) with *z*-score threshold of -1 and 1 by following Gevaert et al. (2006). For pathological images, the whole-slide images with ×40 magnification are tiled into overlapping 1000 × 1000 pixels by adopting bftools in the open microscopy environment (Sun *et al.*, 2018a,b; Yu *et al.*, 2016). Next, we select the 10 densest images of each image series ranked by image density defined as the percentage of non-white (all of the red, green and blue values are below 200 in the 24-bit RGB color space) pixels by following previous study (Yu *et al.*, 2016). Moreover, according to Sun et al. (2018a,b), by using CellProfiler we obtain 2343 image features from pathological images, which contain nucleus size, shape, distribution of pixel intensity and texture of nuclei. The aforementioned normalization and discretization steps are also applied to image features to mitigate the heterogeneity gap between different modalities. After that, the pre-processed genomic and pathological image features are fed into the FSelector package (Cheng *et al.*, 2012) implemented by R, respectively, which performs automatic feature selection from the input by ranking informative features. Finally, from the results of FSelector we choose the top 32 genomic and pathological image features, respectively, for further study (Sun *et al.*, 2018a,b; Yu *et al.*, 2016).

### 2.2 GPDBN

**Fig. 1.**
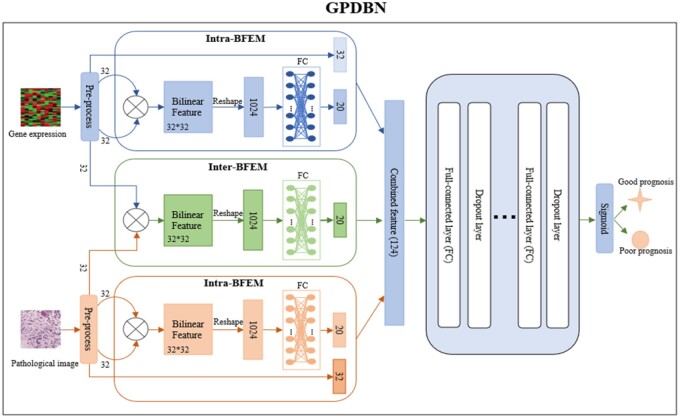
Illustration of the proposed GPDBN framework

The workflow of GPDBN is shown in Figure 1.Inter-modality bilinear features are firstly generated by Inter-BFEM to model complex inter-modality relations for fully exploiting intrinsic relationship of the features derived from genomic data and pathological images. Meanwhile, intra-modality bilinear features within each modality that are beneficial to prognosis prediction are also extracted by Intra-BFEMs. Finally, the intra- and inter-modality bilinear features are combined by multi-layer deep neural network to take advantage of the complementary information between intra-modality and inter-modality relations. The following subsections describe GPDBN in detail.

#### 2.2.1 Inter-BFEM

Previous deep learning-based studies for cancer prognosis prediction have used direct feature combination (Mobadersany *et al.*, 2018; Huang *et al.*, 2019) or score level fusion (Sahasrabudhe *et al.*, 2020; Sun *et al.*, 2018a,b) to integrate data from different modalities, which may not be sufficient to capture the complex inter-modality relations. To address this issue, in GPDBN we propose Inter-BFEM that is inspired by recent achievements in bilinear model (Hou *et al.*, 2019; Yu *et al.*, 2017). It is of note that this idea is similar to the recent work by Chen et al. (2020a,b) in modeling inter-modality relations between genomic data and pathological images for diagnosis and prognosis of glioma. Specifically, given L-dimensional input vectors $g,\;p\in \;R^{L\times 1}$ derived from genomic data and pathological images, respectively, Inter-BFEM utilizes a bilinear function of g and p via fully connected layer followed by ReLU as the activation function. Accordingly, the K-dimensional inter-modality bilinear feature $f^{\mathrm{inter}}\in R^{K\times 1}$ generated by Inter-BFEM can be formulated as:

(1) $$f_{k}^{\mathrm{inter}}=\;\mathrm{ReLU}(W_{k}vec\left(gp^{T}\right)+\;b_{k})$$

where vec(.) is the vectorization operator that converts a matrix to a vector, $f_{k}^{\mathrm{inter}}$ is the k-th value of $f^{\mathrm{inter}}$, $W_{k}$ and $b_{k}$ are learnable weight matrix and bias term of Inter-BFEM, respectively. Also, Eq. (1) can be written by the general form of bilinear model (Tenenbaum and Freeman, 2000):

(2) $$f_{k}^{\mathrm{inter}}=\mathrm{ReLU}(\sum_{i=1}^{L}\sum_{j=1}^{L}w_{\mathrm{ijk}}g_{i}p_{j}+\;b_{k})$$

where $w_{\mathrm{ijk}}$ is the entry of $W_{k}$, $g_{i}$ and $p_{j}$ are the i-th, j-th value of g and p, respectively. Notice that the number of corresponding parameters of Inter-BFEM is K×(L×L+1), which induce substantial computational burdens and require a huge amount of training data to fit when K and L are both large. Therefore, K and L are set to 20 and 32, respectively, in this study.

#### 2.2.2 Intra-BFEMs

Besides inter-modality relations, there are also relations within each modality that may play a crucial role in prognosis prediction. For example, genomic data encompasses all ongoing biological processes in a cell or tissue, where multiple factors do not work independently of each other but are intertwined in a complex and entangled fashion (Chen *et al.*, 2020a,b). Therefore, in GPDBN two Intra-BFEMs are developed to capture intra-modality relations within genomic and image features, respectively. Specifically, intra-modality bilinear features denoted as $f^{\mathrm{intra}\_g},f^{\mathrm{intra}\_p}\in R^{M\times 1}$ are extracted as follows:

(3) $$f_{m}^{\mathrm{intra}\_g}=\mathrm{ReLU}\left(W_{m}^{g}vec\left(gg^{T}\right)+b_{m}^{g}\right)=\mathrm{ReLU}\;(\sum_{i=1}^{L}\sum_{j=1}^{L}w_{\mathrm{ijm}}^{g}g_{i}g_{j}+\;b_{m}^{g})$$

 

(4) $$f_{m}^{\mathrm{intra}\_p}=\mathrm{ReLU}\left(W_{m}^{p}vec\left(pp^{T}\right)+b_{m}^{p}\right)=\mathrm{ReLU}\;(\sum_{i=1}^{L}\sum_{j=1}^{L}w_{\mathrm{ijm}}^{p}p_{i}p_{j}+\;b_{m}^{p})$$

where $W_{m}^{g}$ and $b_{m}^{g}$ are the weight matrix and bias term of Intra-BFEM for genomic features, $w_{\mathrm{ijm}}^{g}$ is the entry of $W_{m}^{g}$, $W_{m}^{p}$ and $b_{m}^{p}$ are the weight matrix and bias term of Intra-BFEM for pathological image features, $w_{\mathrm{ijm}}^{p}$ is the entry of $W_{m}^{p}$, M is the dimension of intra-modality bilinear features and is set to 20 in this study. Accordingly, the output vectors of Intra-BFEMs for genomic and pathological image features are denoted as ${\hat{f}}^{\mathrm{intra}\_g},{\hat{f}}^{\mathrm{intra}\_p}\in R^{L+M}$ and can be defined as follows:

(5) $${\hat{f}}^{\mathrm{intra}\_g}=g⊕f^{\mathrm{intra}\_g}$$

 

(6) $${\hat{f}}^{\mathrm{intra}\_p}=p⊕f^{\mathrm{intra}\_p}$$

where⊕denotes concatenation of vectors.

#### 2.2.3 Prognosis prediction

Considering the intra-modality relations are complementary to the inter-modality relations, GPDBN adopts a multi-layer deep neural network that takes combined features $h\in R^{K+2M+2L}$ as input to improve the predictive performance of breast cancer prognosis, and h can be defined as follows:

(7) $$h={\hat{f}}^{\mathrm{intra}\_g}⊕f^{\mathrm{inter}}⊕\;{\hat{f}}^{\mathrm{intra}\_p}.$$

The multi-layer deep neural network consists of an input layer, S fully connected layers and an output layer. The abstract features of the last fully connected layer are fed into the output layer to generate the final predictive scores of shorter-term and longer-term survivors. To realize the non-linear transformation, we employ ReLU and softmax as the activation functions for the fully connected layers and output layer, respectively. Mathematically, it can be described as follows:

(8) $$o_{1}=\mathrm{ReLU}\left(W_{1}h+b_{1}\right),$$

 

(9) $$o_{s}=\mathrm{ReLU}\left(W_{s}o_{s-1}+b_{s}\right),\;2\leq s\leq S,$$

 

(10) $$P\left(y=1|g,p\right)=\mathrm{softmax}\left(W_{S+1}o_{S}+b_{S+1}\right),$$

 

(11) $$P\left(y=0|g,p\right)=1-P\left(y=1|g,p\right)$$

where S is set to 4, $W_{s},\;b_{s}$ and $o_{s}$ refer to parameter matrices, bias item and output of the s-th fully connected layer, respectively, y represents the corresponding prognosis label. The hyperparameters of the multi-layer deep neural network are listed in Table 1.

**Table 1.** Details of multi-layer deep neural network

	Layer	Details	Output size
Multi-layer deep neural network	Input combined features	concatenate the output of Inter-BFEM and Intra-BFEMs as input	(124)
	Fully connected layer (+ReLU)	500 neurons	(500)
	Dropout layer	*P* = 0.3	(500)
	Fully connected layer (+ReLU)	256 neurons	(256)
	Dropout layer	*P* = 0.3	(256)
	Fully connected layer (+ReLU)	128 neurons	(128)
	Dropout layer	*P* = 0.1	(128)
	Fully connected layer (+ReLU)	32 neurons	(32)
	Dropout layer	*P* = 0.1	(32)
	Softmax output unit	2 neurons	(2)

### 2.3 Training

GPDBN is a unified learning framework and is trained to classify breast cancer patients into two classes: longer-term survivors or shorter-term survivors (Gevaert *et al.*, 2006; Nguyen *et al.*, 2013). Accordingly, Inter-BFEM, Intra-BFEMs and the multi-layer deep neural network in GPDBN are trained with a binary cross entropy objective function defined as follows:

(12) $$L=\;-\frac{1}{N}\sum_{n=1}^{N}{[y}^{n}lnP\left(y^{n}=1|g^{n},p^{n}\right)+\;\left(1-y^{n}\right)ln\left(1-P\left(y^{n}=1|g^{n},p^{n}\right)\right)],$$

where $g^{n},p^{n}$ represent input vectors of the nth training sample, $y^{n}$ represents its corresponding longer-term or shorter-term class label and N is the total number of patients in training set. The weights and biases of fully connected layers in Inter-BFEM, Intra-BFEMs and the multi-layer neural network in GPDBN are parameters to be estimated. We train the model with Adam optimizer that is a widely used stochastic gradient descent algorithm. Meanwhile, mini-batch training strategy is adopted in this study by randomly dividing small proportions of the training samples in each iteration to optimizer loops. We preset learning rate to 4e-4, the number of training epochs to 150 and the optimal value for the batch size adjusted on the cross-validation set is 16. We use Keras, a high-level neural network API written in python, to implement GPDBN under Linux with CPU Intel Xeon 4110 @ 2.10 GHz, GPU NVIDIA GeForce RTX 2080 Ti, and 192GB of RAM.

### 2.4 Evaluation metrics

To evaluate the performance of our proposed GPDBN, in this study we employ several commonly used metrics, containing sensitivity (Sn), specificity (Sp), overall accuracy (Acc), precision (Pre) and F1 scores. The detailed definitions are:

(13) $$Sn=\frac{TP}{TP+FN}$$

 

(14) $$Sp=\frac{TN}{TP+FP}$$

 

(15) $$\mathrm{Acc}=\;\frac{TP+TN}{TP+TN+FP+FN}$$

 

(16) $$\mathrm{Pre}=\;\frac{TP}{TP+FP}$$

 

(17) $$F1=\frac{2\times \mathrm{Pre}\times Sn}{\mathrm{Pre}+Sn}$$

where TP, TN, FP and FN refer to true positives, true negatives, false positives and false negatives, respectively. Furthermore, we apply area under the ROC curve (AUC) and concordance index (C-index) as described in Sun *et al.* (2018a,b) to assess the overall performance. C-index is widely applied to assess the performance of prognosis prediction methods, which quantifies the ranking quality of rankings and is valued from 0 to 1. C-index = 0.5 suggests the model makes ineffective prediction and a higher C-index > 0.5 suggests a better prognosis method. All of the metrics are evaluated on the test splits of the 5-fold cross validation. Specifically, we concatenate the predictive scores from all of the test splits in the 5-fold cross validation and calculate the evaluation metrics and plot ROC curves.

## 3 Results

### 3.1 Performance evaluation of GPDBN

To verify the effectiveness of our proposed GPDBN in contributing to prognosis prediction of breast cancer, we conduct ablation study comparing different model configurations in 5-fold cross validation. First, we train four unimodal models to evaluate the performance of Intra-BFEMs: (i) BaselineG: a multi-layer deep neural network using genomic features as input, (ii) Intra-BFEMG: an Intra-BFEM using genomic features as input, followed by a multi-layer deep neural network, (iii) BaselineP: a multi-layer deep neural network using pathological image features as input, (iv) Intra-BFEMP: an Intra-BFEM using pathological image features as input, followed by a multi-layer deep neural network. After that, we train three multimodal models to evaluate the performance of Inter-BFEM: (i) BaselineGP: a multi-layer deep neural network using directly concatenated genomic and pathological image features as input, (ii) Inter-BFEM*: an Inter-BFEM using genomic and pathological image features as input, followed by a multi-layer deep neural network, (iii) GPDBN: our proposed deep bilinear network using genomic and pathological image features as input. It is of note that all aforementioned multi-layer deep neural networks have the same hyperparameters as described in Table 1.

**Fig.2.**
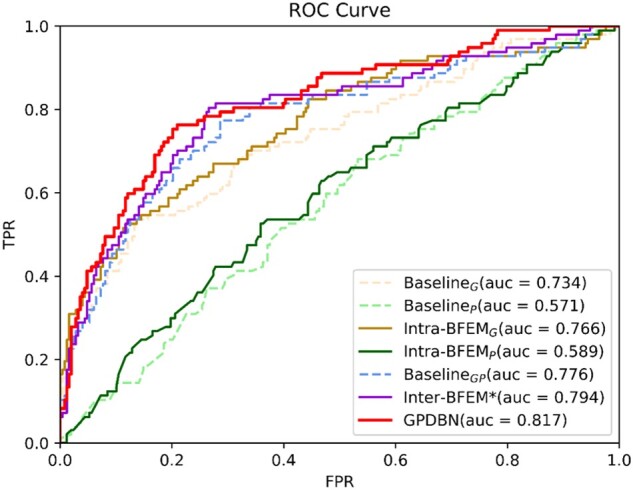
ROC curves of GPDBN for breast cancer prognosis prediction

**Fig. 3.**
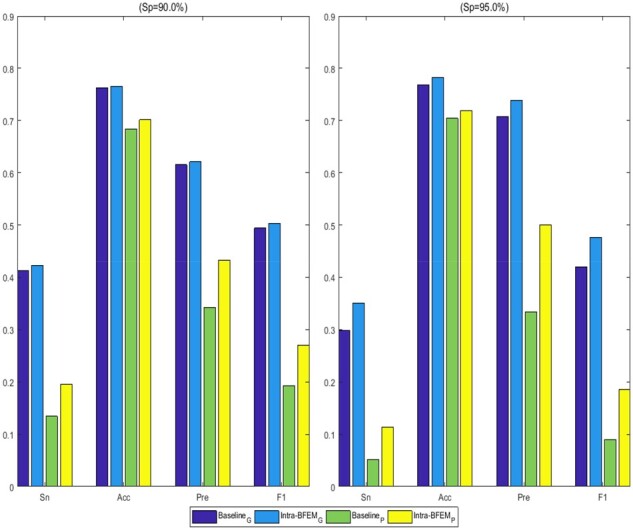
The values of Sn, Acc, Pre and F1 of GPDBN for breast cancer prognosis prediction at stringent levels of Sp = 90.0% (left) and Sp = 95.0% (right)

Table 2 shows the C-index and AUC values of different models. We can find that Intra-BFEM_G_ and Intra-BFEM_P_ achieve consistently better overall performance than Baseline_G_ and Baseline_P_, respectively. For example, the C-index and AUC values of Intra-BFEM_G_ are 0.695 and 0.779, while the corresponding C-index and AUC values of Baseline_G_ are 0.674 and 0.734, respectively. It is of note that the average C-index and AUC values over 5-fold cross validation (Supplementary Table S1) show similar performance. Besides, we plot ROC curves to compare the predictive performance in Figure 2, which suggest that our proposed Intra-BFEMs have great efficiency on both genomic data and pathological images. Furthermore, we calculate the corresponding Sn, Acc, Pre and F1 of all compared methods with Sp equal to 90.0% or 95.0% and the histograms of these methods are shown in Figure 3. From these results, we can see that when Sp is equal to 95.0%, by adopting Intra-BFEM Sn is improved by 6.2% and 5.1% on pathological images and genomic data, respectively. Meanwhile, compared with Baseline_P_ and Baseline_G_, the Pre values of Intra-BFEM_P_ and Intra-BFEM_G_ have an improvement of 16.7% and 3.2% respectively. The above results verify the advantage of using Intra-BFEMs for prognosis prediction of breast cancer in our proposed GPDBN framework by capturing the intra-modality relations within genomic data and pathological images.

**Table 2.** C-index and AUC values of GPDBN for breast cancer prognosis prediction

Data	Method	C-index	AUC
Genomic data	Baseline_G_	0.674	0.734
	Intra-BFEM_G_	0.695	0.779
Pathological images	Baseline_P_	0.569	0.571
	Intra-BFEM_P_	0.578	0.585
Genomic data + Pathological images	Baseline_GP_	0.703	0.775
	Inter-BFEM*	0.708	0.793
	GPDBN	**0.723**	**0.817**

The bold values show the performances of proposed GPDBN method.

**Fig. 4.**
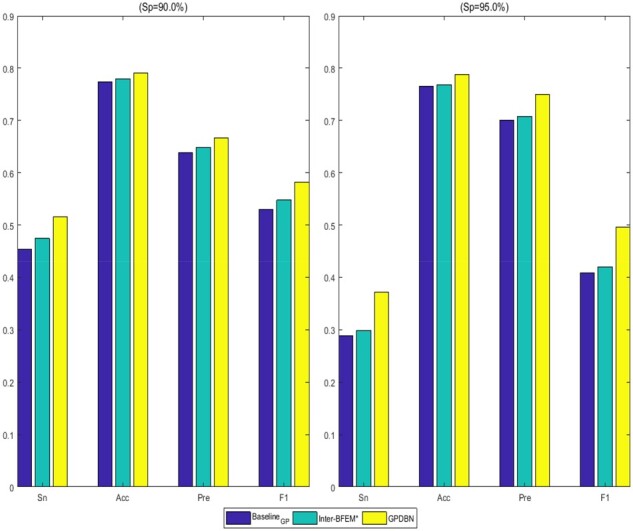
The values of Sn, Acc, Pre and F1 of different methods for breast cancer prognosis prediction at stringent levels of Sp = 90.0% (left) and Sp = 95.0% (right)

As indicated in Table 2, Intra-BFEM_G_ outperforms Intra-BFEM_P_ with an improvement of more than 11.2% in C-index, which reflects the importance of genomic data for prognosis prediction. Although the prognostic performance on pathological images is worse than genomic data, we can also see its effect in improving performance when integrated with genomic data. For example, the C-index value of Baseline_GP_ is 0.703, which has 13.4% and 2.9% improvement over Baseline_P_ and Baseline_G_, respectively. Meanwhile, we also find that Inter-BFEM* can obtain higher C-index and AUC values than Baseline_GP_. For example, the AUC value achieved by Inter-BFEM* is 0.793, compared with 0.775 obtained by Baseline_GP_. This demonstrates the power of Inter-BFEM in capturing complex inter-modality relations for prognosis prediction. More importantly, by adopting Intra-BFEM and Inter-BFEM simultaneously, GPDBN obtains the best performance with remarkable improvements on both C-index and AUC values in comparison with other methods. For example, the C-index value achieved by GPDBN is 0.723, which has an improvement of 2.0% and 1.5% over Baseline_GP_ and Inter-BFEM*, respectively. Furthermore, we calculate the Sn, Acc, Pre and F1 of all compared methods, and the histograms these methods are displayed in Figure 4. From these results, we can find that GPDBN consistently yields the best performance on all metrics. Taken together, these results demonstrate that our proposed GPDBN can effectively integrate genomic data and pathological images by taking advantage of the complementary information between inter-modality and intra-modality relations obtained by Inter-BFEM and Intra-BFEM, respectively.

### 3.2 Comparison with existing methods

To further evaluate the performance of our proposed GPDBN, we compare it with several existing methods, namely BoostCI, PCRM, superPC, En-Cox, LASSO-Cox, MDNNMD and DeepCorrSurv, and the C-index values of different methods are listed in Table 3. It is obvious that all these methods have satisfying performance by using genomic data. For example, the C-index values obtained by BoostCI and LASSO-Cox are 0.677 and 0.690, respectively, meanwhile deep learning-based MDNNMD and GPDBN have similar results of 0.691 and 0.695, respectively. These results further corroborate the conclusion that genomic data plays a crucial role in prognosis prediction of breast cancer. By using pathological images, we can find that deep learning-based approaches generally exhibit better performance than other methods evaluated in this study. For example, with a C-index value of 0.570, MDNNMD outperforms all other non-deep learning methods except superPC. At the same time, GPDBN achieves the best performance among all investigated methods and obtains a C-index improvement of 1.3% compared with MDNNMD, which indicates the effectiveness of GPDBN in breast cancer prognosis prediction.

**Table 3.** Performance comparison of the proposed GPDBN and other methods using C-index value

	Genomic data	Pathological images	Genomic data + pathological images
BoostCI	0.677	0.435	0.681
PCRM	0.569	0.569	0.688
superPC	0.696	0.573	0.696
En-Cox	0.688	0.560	0.697
LASSO-Cox	0.690	0.564	0.700
MDNNMD	0.691	0.570	0.704
DeepCorrSurv	NA	NA	0.694
GPDBN	0.695	0.583	0.723

**Fig. 5.**
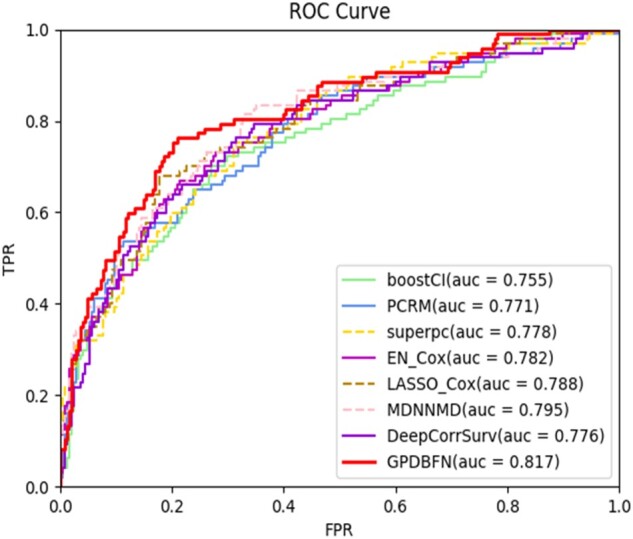
ROC curves of GPDBN and other methods by employing both genomic data and pathological images

From Table 3, we can also observe that compared with using single-modality data alone, the application of both genomic data and pathological images enhances the performance for most of the methods. For example, the C-index value of LASSO-Cox using both genomic data and pathological images is increased by 1.0% and 13.6% compared with those obtained by using only genomic data and pathological images, respectively. These suggest that pathological images can provide valuable predictive information additional to those provided by genomic data. Importantly, GPDBN outperforms all non-deep learning methods with remarkable improvement in C-index value, which indicates that sophisticated deep learning-based methods can be advantageous in integrating data from different modalities. At the same time, GPDBN achieves the highest C-index value of 0.723 among all the deep learning-based methods, which demonstrates that compared with MDNNMD and DeepCorrSurv, the proposed GPDBN can efficiently integrate genomic data and pathological images. In addition to C-index value, the ROC curves of different methods using both genomic data and pathological images are plotted in Figure 5, in which GPDBN also achieves the best AUC value of 0.817 among all investigated methods. Taken together, these results suggest that our proposed GPDBN can take advantage of not only inter- but also intra-modality relations to significantly improve the performance of breast cancer prognosis prediction. Moreover, we conduct survival analysis on TCGA breast cancer dataset, in which GPDBN is supervised with the Cox objective function (Chen *et al.*, 2020a,b) and the detailed information is provided in Supplementary Methods and Supplementary Table S2. As shown in Supplementary Table S3, we can find that the average C-index value of GPDBN compares favorably with Pathomic Fusion (Chen *et al.*, 2020a,b) for survival analysis.

**Fig. 6.**
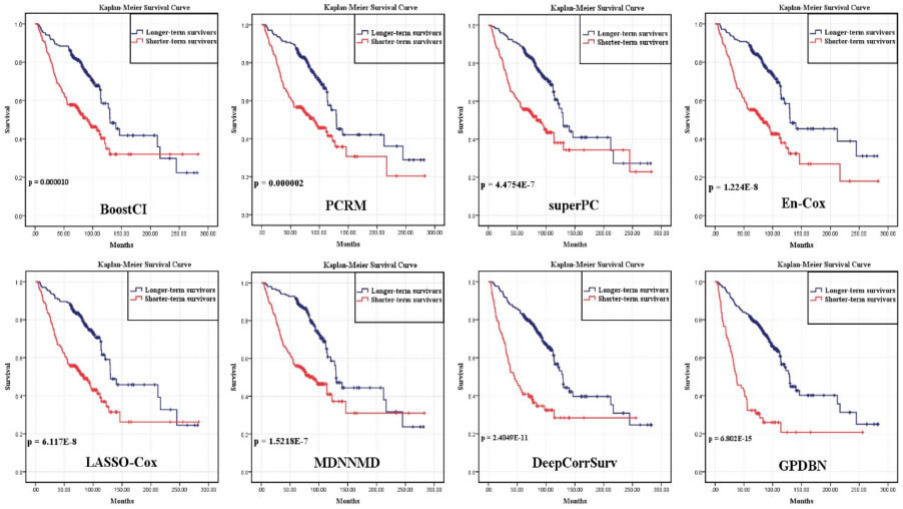
Performance comparison of the proposed GPDBN and other methods using Kaplan–Meier curve

To further evaluate the performance of GPDBN, Kaplan-Meier curves of different methods are plotted and displayed in Figure 6. We can observe that for non-deep learning-based methods, LASSO-Cox provides better prognostic prediction with a log-rank test P value of 6.117E-8 than other methods such as BoostCI (log-rank test P value = 0.000010) and superPC (log-rank test P value = 4.4754E-7). In comparison with these non-deep learning methods, deep learning-based DeepCorrSurv provides competitive performance (log-rank test P value = 2.4049E-11) by considering intrinsic relationship of the features across different modalities in deep learning architecture. Moreover, by effectively integrating genomic data and pathological images, our proposed GPDBN can successfully distinguish shorter-term survivors from longer-term survivors and achieve superior performance (log-rank test P value = 6.802E-15) over all other methods. In addition, Kaplan-Meier curves of GPDBN supervised with the Cox objective function and Pathomic Fusion are plotted in Supplementary Figures S1 and S2, and GPDBN provides better survival prediction with a log-rank test *P* value of 2.0452E-10 than Pathomic Fusion (log-rank test *P* value = 0.000117). These results further confirm the effectiveness of GPDBN in breast cancer prognosis prediction.

### 3.3 Visualization of features

**Fig. 7.**
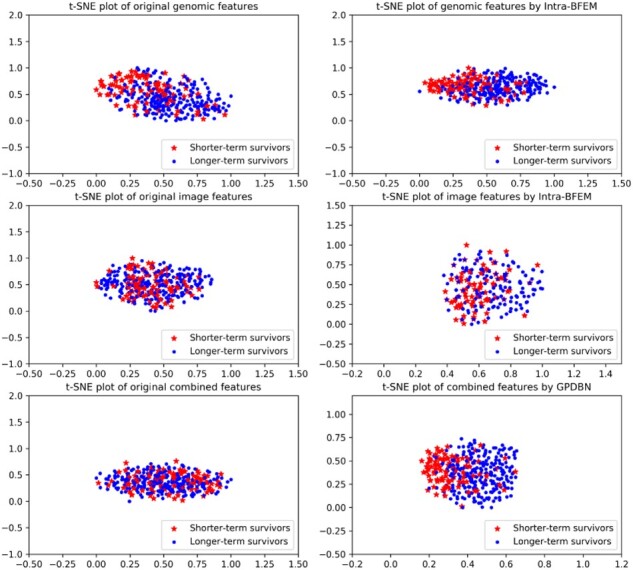
Visualization of original genomic, pathological image, combined features and the corresponding abstract features extracted by our proposed method. The red star represents shorter-term survivors, and the blue dot represents longer-term survivors

To visualize the superiority of the proposed method in prognosis prediction, a popular algorithm t-SNE is used to squeeze abstract features extracted by Intra-BFEM and GPDBFN into a 2-D space. As shown in Figure 7, original genomic and pathological image features are overlapped in mixture, while combined genomic and pathological image features show separate trends, which suggests that the integration of data from different modalities can contribute to more accurate prognosis prediction of breast cancer. Furthermore, we can observe that compared with using original genomic and pathological image features, using abstract features extracted by Intra-BFEM shows an obvious improvement in separating shorter-term survivors and longer-term survivors, which become even more evident when using combined features extracted by GPDBFN. These results show that original genomic and pathological image features can be transformed into meaningful representations by Intra-BFEM, respectively, and GPDBFN can generate better combined representation with stronger discriminant power in distinguishing shorter-term and longer-term survivors.

## 4 Discussion

In this study, we propose a novel deep learning-based GPDBN framework to effectively integrate both genomic data and pathological images for more accurate breast cancer prognosis prediction. Our findings suggest that prognosis prediction methods based on data from different modalities outperform those using single-modality data and our proposed Intra-BFEM and Inter-BFEM can efficiently capture complex relations not only within each modality but also between different modalities. Meanwhile, the comprehensive experimental results show that by using both intra- and inter-modality bilinear features as input, GPDBN compares favorably with many existing methods. Furthermore, the visualization results also indicate its powerful capability in distinguishing shorter-term survivors from longer-term survivors. The main contributions of this work are as follows: (i) we design Inter-BFEM to generate inter-modality bilinear features that can fully exploit intrinsic relationship of the features across different modalities, (ii) two efficient deep bilinear encoding modules named Intra-BFEMs are proposed to calculate intra-modality bilinear features for capturing relations within each modality that are also beneficial for breast cancer prognosis prediction, (iii) by taking advantage of the complementary information between intra-modality and inter-modality relations, the proposed GPDBN shows great strength in integrating data from different modalities, and achieves remarkable performance for predicting the prognosis of breast cancer.

Although GPDBN has enhanced the predictive performance of breast cancer prognosis, there is still considerable room for further expansion and improvement. Firstly, limited by available genomic data and pathological images, this work can further improve prognostic performance by introducing more breast cancer patients. Secondly, other genomic data (e.g. copy number, miRNA expression) have also been reported to be beneficial for cancer prognosis prediction (Huang *et al.*, 2019). To integrate more modalities, computational approaches such as tensor fusion (Chen *et al.*, 2020a,b; Zadeh *et al.*, 2017) can be incorporated into the proposed GPDBN framework in future work. Meanwhile, other computationally efficient bilinear models (Fukui *et al.*, 2016; Gao *et al.*, 2016) can also be explored in the future to reduce memory consumption and computation times when more modalities are adopted. Thirdly, it is possible to further improve our framework by building a pancancer model for prognosis that also predicts prognosis of breast cancer (Cheerla and Gevaert, 2019). Finally, it is important to develop deep learning-based prognosis prediction method with improved interpretability (Ma *et al.*, 2018). In conclusion, we present a novel deep bilinear network for breast cancer prognosis prediction, which has the potential to be extended to integrate data from different modalities for other predictive tasks and provides clues for further cancer prognosis research.

## Funding

This work was supported by the National Natural Science Foundation of China [61971393, 61871361, 61571414, 61471331].

*Conflict of Interest*: none declared.

## Supplementary Material

btab185_Supplementary_Data

## References

[btab185-B1] Cheerla A. , Gevaert O. (2019) Deep learning with multimodal representation for pancancer prognosis prediction. Bioinformatics, 35, i446–i454. 31510656 10.1093/bioinformatics/btz342 PMC6612862

[btab185-B2] Chen R. et al (2020a) Deep-learning approach to identifying cancer subtypes using high-dimensional genomic data. Bioinformatics, 36, 1476–1483. 31603461 10.1093/bioinformatics/btz769 PMC8215925

[btab185-B3] Chen R.J. et al (2020b) Pathomic fusion: an integrated framework for fusing histopathology and genomic features for cancer diagnosis and prognosis. IEEE Trans. Med. Imag., 99, 1–1. 10.1109/TMI.2020.3021387 PMC10339462 32881682

[btab185-B4] Cheng J. et al (2018) Identification of topological features in renal tumor microenvironment associated with patient survival. Bioinformatics, 34, 1024–1030. 29136101 10.1093/bioinformatics/btx723 PMC7263397

[btab185-B5] Cheng J. et al (2017) Integrative analysis of histopathological images and genomic data predicts clear cell renal cell carcinoma prognosis. Cancer Res., 77, e91–e100. 29092949 10.1158/0008-5472.CAN-17-0313 PMC7262576

[btab185-B6] Cheng T. et al (2012) FSelector: a Ruby gem for feature selection. Bioinformatics, 28, 2851–2852. 22942017 10.1093/bioinformatics/bts528 PMC3476337

[btab185-B7] Ching T. et al (2018) Cox-nnet: an artificial neural network method for prognosis prediction of high-throughput omics data. PLoS Comput. Biol., 14, e1006076. 29634719 10.1371/journal.pcbi.1006076 PMC5909924

[btab185-B8] Courtiol P. et al (2019) Deep learning-based classification of mesothelioma improves prediction of patient outcome. Nat. Med., 25, 1519–1525. 31591589 10.1038/s41591-019-0583-3

[btab185-B9] Ding Z. et al (2016) Evaluating the molecule-based prediction of clinical drug responses in cancer. Bioinformatics, 32, 2891–2895. 27354694 10.1093/bioinformatics/btw344

[btab185-B10] Fukui A. et al (2016) Multimodal compact bilinear pooling for visual question answering and visual grounding. In *Proceedings of the 2016 Conference on Empirical Methods in Natural Language Processing*, pp. 457–468. Austin, Texas, USA.

[btab185-B11] Gao P. et al (2019) Dynamic fusion with intra-and inter-modality attention flow for visual question answering. In *Proceedings of the IEEE/CVF Conference on Computer Vision and Pattern Recognition*, pp. 6639–6648. Long Beach, CA, USA.

[btab185-B12] Gao Y. et al (2016) Compact bilinear pooling. In *Proceedings of the IEEE conference on computer vision and pattern recognition*, pp. 317–326. Las Vegas, NV, USA.

[btab185-B13] Gevaert O. et al (2006) Predicting the prognosis of breast cancer by integrating clinical and microarray data with Bayesian networks. Bioinformatics, 22, e184–e190. 16873470 10.1093/bioinformatics/btl230

[btab185-B14] Hortobagyi G.N. et al; ABREAST Investigators. (2005) The global breast cancer burden: variations in epidemiology and survival. Clin. Breast Cancer, 6, 391–401. 16381622 10.3816/cbc.2005.n.043

[btab185-B15] Hou M. et al (2019) Deep multimodal multilinear fusion with high-order polynomial pooling. Adv. Neural Inf. Process. Syst.,32, 12136–12145.

[btab185-B16] Huang Z. et al (2019) SALMON: survival analysis learning with multi-omics neural networks on breast cancer. Front. Genet., 10, 166. 30906311 10.3389/fgene.2019.00166 PMC6419526

[btab185-B17] Ma J. et al (2018) Using deep learning to model the hierarchical structure and function of a cell. Nat. Methods, 15, 290–298. 29505029 10.1038/nmeth.4627 PMC5882547

[btab185-B18] Mobadersany P. et al (2018) Predicting cancer outcomes from histology and genomics using convolutional networks. Proc. Natl. Acad. Sci. USA, 115, E2970–E2979. 29531073 10.1073/pnas.1717139115 PMC5879673

[btab185-B19] Moon W.K. et al (2017) Computer-aided prediction of axillary lymph node status in breast cancer using tumor surrounding tissue features in ultrasound images. Comput. Methods Programs Biomed., 146, 143–150. 28688484 10.1016/j.cmpb.2017.06.001

[btab185-B20] Ngiam J. et al (2011) Multimodal deep learning. In *Proceedings of the 28th International Conference on Machine Learning*, pp. 689–696. Bellevue, WA, USA.

[btab185-B21] Nguyen C. et al (2013) Random forest classifier combined with feature selection for breast cancer diagnosis and prognostic. Journal of B*iomedical Science & Engineering*, 06(5), 551–560.

[btab185-B22] Ning Z. et al (2020) Integrative analysis of cross-modal features for the prognosis prediction of clear cell renal cell carcinoma. Bioinformatics, 36, 2888–2895. 31985775 10.1093/bioinformatics/btaa056

[btab185-B23] Reis-Filho J.S. , Pusztai L. (2011) Gene expression profiling in breast cancer: classification, prognostication, and prediction. Lancet, 378, 1812–1823. 22098854 10.1016/S0140-6736(11)61539-0

[btab185-B24] Sahasrabudhe M. et al (2020) Deep multi-instance learning using multi-modal data for diagnosis of lymphocytosis. IEEE J. Biomed. Health Inf., 99, 1–1. 10.1109/JBHI.2020.3038889 33206611

[btab185-B25] Shao W. et al (2019) Integrative analysis of pathological images and multi-dimensional genomic data for early-stage cancer prognosis. IEEE Trans. Med. Imag., 39, 99–110. 10.1109/TMI.2019.2920608 31170067

[btab185-B26] Shao W. et al (2020) Multi-task multi-modal learning for joint diagnosis and prognosis of human cancers. Med. Image Anal., 65, 101795. 32745975 10.1016/j.media.2020.101795

[btab185-B27] Sun D. et al (2018a) Integrating genomic data and pathological images to effectively predict breast cancer clinical outcome. Comput. Methods Programs Biomed., 161, 45–53. 29852967 10.1016/j.cmpb.2018.04.008

[btab185-B28] Sun D. et al (2018b) A multimodal deep neural network for human breast cancer prognosis prediction by integrating multi-dimensional data. IEEE/ACM Trans. Comput. Biol. Bioinf., 16, 841–850. 10.1109/TCBB.2018.2806438 29994639

[btab185-B29] Tenenbaum J.B. , Freeman W.T. (2000) Separating style and content with bilinear models. Neural Comput., 12, 1247–1283. 10935711 10.1162/089976600300015349

[btab185-B30] Van De Vijver M.J. et al (2002) A gene-expression signature as a predictor of survival in breast cancer. N. Engl. J. Med., 347, 1999–2009. 12490681 10.1056/NEJMoa021967

[btab185-B31] Wang Y. et al (2005) Gene-expression profiles to predict distant metastasis of lymph-node-negative primary breast cancer. Lancet, 365, 671–679. 15721472 10.1016/S0140-6736(05)17947-1

[btab185-B32] Xu J. et al (2016) Stacked sparse autoencoder (SSAE) for nuclei detection on breast cancer histopathology images. IEEE Trans. Med. Imag., 35, 119–130. 10.1109/TMI.2015.2458702 PMC4729702 26208307

[btab185-B33] Xu X. et al (2012) A gene signature for breast cancer prognosis using support vector machine. In: *2012 5th International Conference on BioMedical Engineering and Informatics*, pp. 928–931. Chongqing, China.

[btab185-B34] Yao J. et al (2017) Deep correlational learning for survival prediction from multi-modality data. In: *International Conference on Medical Image Computing and Computer-Assisted Intervention*, pp. 406–414. Quebec City, QC, Canada.

[btab185-B35] Yu K.-H. et al (2016) Predicting non-small cell lung cancer prognosis by fully automated microscopic pathology image features. Nat. Commun., 7, 1–10. 10.1038/ncomms12474 PMC4990706 27527408

[btab185-B36] Yu Z. et al (2017) Multi-modal factorized bilinear pooling with co-attention learning for visual question answering. In *Proceedings of the IEEE International Conference on Computer Vision*, pp. 1821–1830. Venice, Italy.

[btab185-B37] Yuan Y. et al (2012) Quantitative image analysis of cellular heterogeneity in breast tumors complements genomic profiling. Sci. Transl. Med., 4, 157ra143–157ra143. 10.1126/scitranslmed.3004330 23100629

[btab185-B38] Zadeh A. et al (2017) Tensor fusion network for multimodal sentiment analysis. In *Proceedings of the 2017 Conference on Empirical Methods in Natural Language Processing*, pp. 1114–1125. Copenhagen, Denmark.

[btab185-B39] Zhu X. et al (2016) Deep convolutional neural network for survival analysis with pathological images. In: *2016 IEEE International Conference on Bioinformatics and Biomedicine (BIBM)*, pp. 544–547. Shenzhen, China.

[btab185-B40] Zhu Y. et al (2014) TCGA-assembler: open-source software for retrieving and processing TCGA data. Nat. Methods, 11, 599–600. 24874569 10.1038/nmeth.2956 PMC4387197

