# Supplementary material for: GPDBN: deep bilinear network integrating both genomic data and pathological images for breast cancer prognosis prediction
Source: Bioinformatics. 2021 Mar 18;37(18):2963–70. doi: 10.1093/bioinformatics/btab185 (PMC8479662; doi:10.1093/bioinformatics/btab185)
Supplement: btab185_Supplementary_Data [file btab185_supplementary_data.zip › supplementary.docx]

GPDBN: deep bilinear network integrating both genomic data and pathological images for breast cancer prognosis prediction

**Supplementary Material**

Zhiqin Wang, Ruiqing Li, Minghui Wang and Ao Li

**1 Supplementary Methods**

**1.1 Dataset for survival analysis**

Besides using survival status (living less or more than 5 years) in a binary classification problem, we also take survival times (overall survival months) into account for survival analysis. Breast cancer patient samples adopted in this study include matched digital whole-slide images and gene expression profiles, which are acquired from The Cancer Genome Atlas (TCGA) data portal (Zhu*, et al.*, 2014). Patients with missing one or two modality data are excluded and finally 1015 patients are enrolled in this study. The details of the patient information are shown in Table S2. Censored patients mean that the death events of these patients are not observed during their follow up, and their exact survival times are longer than their recorded data, while non-censored category corresponds to the patients whose recorded survival times are the exact time from initial diagnosis to death (Shao*, et al.*, 2019). 5-fold cross validation is conducted on the dataset with 80% of the data in each fold used for training and 20% for testing.

**1.2 Experimental setup for survival analysis**

GPDBN is supervised by the Cox objective function (Chen*, et al.*, 2020) for survival analysis. We train the model with Adam optimizer that is a widely used stochastic gradient descent algorithm. Different from using survival status (living less or more than 5 years) in a binary classification problem, we take survival times (overall survival months) into account. We perform 5-fold cross validation with 1015 patients and the Concordance index (C-index) value of each fold is averaged to evaluate the performance by following previous survival analysis study on breast cancer (Huang*, et al.*, 2019). We compare GPDBN with another survival analysis method Pathomic Fusion (Chen*, et al.*, 2020) using the same experimental setup. Specifically, to make a fair comparison, we use the same 5-folds and the genomic and pathological image features adopted by GPDBN are used as the input of the multimodal tensor fusion via Krocnecker Product and gating-based attention in Pathomic Fusion..

**Reference:**

Chen, R.J.*, et al.* (2020) Pathomic fusion: an integrated framework for fusing histopathology and genomic features for cancer diagnosis and prognosis. *IEEE Transactions on Medical Imaging*. IEEE.doi: 10.1109/TMI.2020.3021387.

Huang, Z.*, et al.* (2019) SALMON: Survival analysis learning with multi-omics neural networks on breast cancer. *Frontiers in genetics*,10,166.

Shao, W.*, et al.* (2019) Integrative analysis of pathological images and multi-dimensional genomic data for early-stage cancer prognosis. *IEEE Transactions on Medical Imaging*,39(1),99-110.

Zhu, Y., Qiu, P. and Ji, Y. (2014) TCGA-assembler: open-source software for retrieving and processing TCGA data. *Nature methods*,11(6),599-600.

**2 Supplementary Figures**


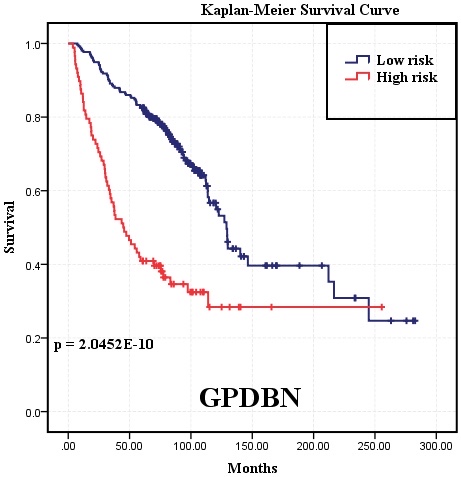


Fig S1. K-M curve of GPDBN for survival analysis

*
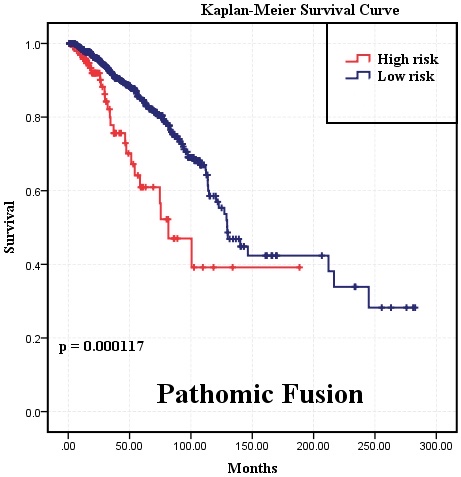
*

Fig S2. K-M curve of Pathomic Fusion for survival analysis

**3 Supplementary Tables**

Table S1. The average C-index and AUC values over 5-fold cross validation for breast cancer prognosis prediction

| Data | Method | C-index | AUC |
| --- | --- | --- | --- |
| Genomic data | Baseline_G_ | 0.685$\pm$0.066 | 0.763$\pm$0.057 |
|  | Intra-BFEM_G_ | 0.688$\pm$0.046 | 0.773$\pm$0.041 |
| Pathological images | Baseline_P_ | 0.564$\pm$0.049 | 0.577$\pm$0.045 |
|  | Intra-BFEM_P_ | 0.572$\pm$0.026 | 0.589$\pm$0.050 |
| Genomic data +  Pathological images | Baseline_GP_ | 0.705$\pm$0.026 | 0.786$\pm$0.009 |
|  | Inter-BFEM* | 0.710$\pm$0.044 | 0.799$\pm$0.035 |
|  | GPDBN | **0.725**$\boldsymbol{\pm}$**0.066** | **0.808**$\boldsymbol{\pm}$**0.053** |

Table S2. Details of TCGA breast cancer dataset for survival analysis

| Total | Censored | Non-Censored |
| --- | --- | --- |
| 1015 | 869 | 146 |

Table S3. C-index comparison of different methods for survival analysis

| Methods | Mean | Std. |
| --- | --- | --- |
| GPDBN-Cox | 0.706 | 0.036 |
| Pathomic Fusion | 0.635 | 0.052 |
