# Supplementary material for: GPDBN: deep bilinear network integrating both genomic data and pathological images for breast cancer prognosis prediction
Source: Bioinformatics. 2021 Mar 18;37(18):2963–70. doi: 10.1093/bioinformatics/btab185 (PMC8479662; doi:10.1093/bioinformatics/btab185)
Supplement: btab185_Supplementary_Data [file btab185_supplementary_data.zip › supplementary figure.docx]

**Supplementary Figures**


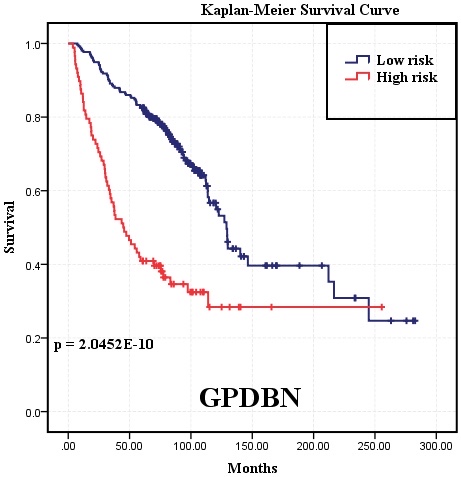


Fig S1. K-M curve of GPDBN for survival analysis

*
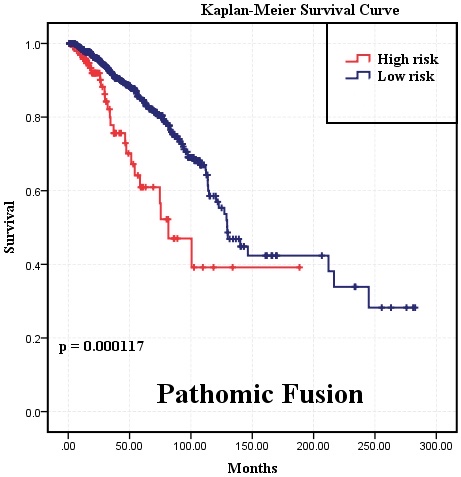
*

Fig S2. K-M curve of Pathomic Fusion for survival analysis
